# Vascular and parenchymal amyloid pathology in an Alzheimer disease knock-in mouse model: interplay with cerebral blood flow

**DOI:** 10.1186/1750-1326-9-28

**Published:** 2014-08-09

**Authors:** Hongmei Li, Qinxi Guo, Taeko Inoue, Vinicia A Polito, Katsuhiko Tabuchi, Robert E Hammer, Robia G Pautler, George E Taffet, Hui Zheng

**Affiliations:** 1 Huffington Center on Aging, Baylor College of Medicine, Houston, Texas 77030, USA; 2 Departments of Neuroscience, University of Texas Southwestern Medical Center at Dallas, Dallas, Texas 75390, USA; 3 Departments of Molecular Physiology and Biophysics, Baylor College of Medicine, Houston, Texas 77030, USA; 4 Departments of Biochemistry, University of Texas Southwestern Medical Center at Dallas, Dallas, Texas 75390, USA; 5 Medicine-Geriatrics and Cardiovascular Sciences, Baylor College of Medicine, Houston, Texas 77030, USA; 6 Molecular and Human Genetics, Baylor College of Medicine, Houston, Texas 77030, USA; 7 Department of Molecular & Cellular Physiology, Shinshu University School of Medicine, Matsumoto, Nagano 290-8621, Japan

**Keywords:** Alzheimer disease, Parenchymal plaque, Cerebral amyloid angiopathy (CAA), Dutch mutation, Cerebral blood flow (CBF), Transverse aortic constriction (TAC)

## Abstract

**Background:**

Accumulation and deposition of β-amyloid peptides (Aβ) in the brain is a central event in the pathogenesis of Alzheimer’s disease (AD). Besides the parenchymal pathology, Aβ is known to undergo active transport across the blood–brain barrier and cerebral amyloid angiopathy (CAA) is a prominent feature in the majority of AD. Although impaired cerebral blood flow (CBF) has been implicated in faulty Aβ transport and clearance, and cerebral hypoperfusion can exist in the pre-clinical phase of Alzheimer’s disease (AD), it is still unclear whether it is one of the causal factors for AD pathogenesis, or an early consequence of a multi-factor condition that would lead to AD at late stage. To study the potential interaction between faulty CBF and amyloid accumulation in clinical-relevant situation, we generated a new amyloid precursor protein (APP) knock-in allele that expresses humanized Aβ and a Dutch mutation in addition to Swedish/London mutations and compared this line with an equivalent knock-in line but in the absence of the Dutch mutation, both crossed onto the PS1M146V knock-in background.

**Results:**

Introduction of the Dutch mutation results in robust CAA and parenchymal Aβ pathology, age-dependent reduction of spatial learning and memory deficits, and CBF reduction as detected by fMRI. Direct manipulation of CBF by transverse aortic constriction surgery on the left common carotid artery caused differential changes in CBF in the anterior and middle region of the cortex, where it is reduced on the left side and increased on the right side. However these perturbations in CBF resulted in the same effect: both significantly exacerbate CAA and amyloid pathology.

**Conclusions:**

Our study reveals a direct and positive link between vascular and parenchymal Aβ; both can be modulated by CBF. The new APP knock-in mouse model recapitulates many symptoms of AD including progressive vascular and parenchymal Aβ pathology and behavioral deficits in the absence of APP overexpression.

## Background

Deposition of β-amyloid peptides (Aβ) derived from the proteolytic processing of the amyloid precursor protein (APP) in the brain is a defining pathological hallmark of Alzheimer’s disease (AD) [1]. Although most AD cases are late-onset, a small percentage of individuals develop early-onset AD due to the presence of autosomal dominant mutations in *APP*, *PSEN1* or *PSEN2.* These familial AD (FAD) mutations are known to alter the production of Aβ, leading to accelerated amyloid pathology. Besides the parenchymal Aβ, cerebral amyloid angiopathy (CAA) is frequently observed in aged human brains and is present in majority of AD and vascular cognitive impairment patients (reviewed by [2]). It is known that Aβ undergoes active transport across the blood–brain barrier (BBB) [3,4]. Accordingly, BBB impairment and/or age-associated reduction of cerebral blood flow (CBF) has been speculated to contribute to faulty Aβ clearance in late-onset AD ( [5] and reviewed by [6]). Of therapeutic importance, pre-clinical and clinical trials of Aβ vaccination have shown to enhance brain to plasma Aβ efflux [7,8], leading to aggravated CAA and microhemorrhages [9-13]. However, contradicting observations were also reported that intraperitoneal administration of antibody 266 retarded Aβ clearance from the brain [14]. While the reason for the different outcomes remains to be established, it argues for the importance to understand the relationship between vascular and parenchymal Aβ, as well as their interactions with cerebral hemodynamics.

Whereas most of the APP FAD mutations lead to early-onset familial AD with variable CAA, the APP E693Q (E22Q of Aβ) mutation is known to cause hereditary cerebral hemorrhage with amyloidosis of Dutch type (HCHWA-D, herein referred to as Dutch) with affected brains exhibiting extensive CAA but limited parenchymal Aβ deposits [15]. Accordingly, various transgenic mouse lines expressing different APP FAD mutations have been created to model AD and/or CAA (http://www.alzforum.org/research-models for current list). However, these animals overexpress transgenes ectopically, which confound the analysis of AD-relevant pathways [16]. Indeed, it has been increasingly recognized that at least some of the phenotypes reported were due to APP overexpression independent of Aβ pathology [17-19].

To facilitate the development of CAA and AD neuropathology in the absence of overexpressing the exogenous APP, we created a new *APP* knock-in mouse that expresses humanized Aβ containing the Dutch mutation in addition to Swedish/London mutations, and compared this line with an equivalent knock-in line but without the Dutch mutation [20,21]. We found that inclusion of the Dutch mutation is associated with age-dependent reduction of CBF, accelerated CAA and Aβ deposition and behavioral deficits. Further, manipulation of CBF by aortic constriction leads to global enhancement of Aβ pathology in both brain parenchyma and cerebral blood vessels, thus revealing a potent and direct regulation of Aβ pathology by CBF.

## Results

### Generation and biochemical analysis of APP DSL knock-in mice

**Figure 1:**
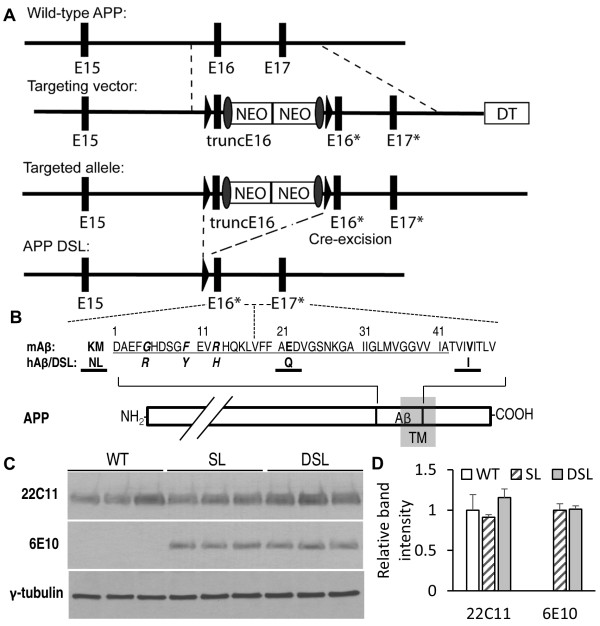
**Generation of APP DSL knock-in mice. A**. Gene targeting strategy to generate the APP DSL knock-in allele. Wild type mouse exon 16 and 17 (E16, E17) were replaced by a truncated exon 16 (truncE16), double neo-cassette, followed by exon 16 with humanized Aβ sequence and the Swedish mutation (E16*), and exon 17 with the Dutch and London mutations (E17*). TruncE16 and the neo-cassette were flanked by two loxP sites, which were removed after cre-excision. DT: diphtheria toxin, which was lost upon homologous recombination. **B**. Schematic diagram of amino acid alterations in APP DSL knock-in line. Aβ42 sequence is marked by thin underline. Residues mutated to humanize Aβ are shown in bold and italic; residues with FAD mutations (Swedish: NL, Dutch: Q, and London: I) are shown in thick-underline. TM: transmembrane region. **C**. Expression of APP is similar in brain lysates of 3-month-old wild type (WT), SL and DSL knock-in lines, as detected by western blot using antibody against the N-terminal region of APP (22C11) or humanized Aβ region (6E10). γ-tubulin was used as loading control. WT: wild type; SL: APP with humanized Aβ region and Swedish and London mutations; DSL: APP with humanized Aβ region and Swedish, Dutch, and London mutations. **D**. Quantification of C.

We replaced exons 16 and 17 of the mouse *APP* gene with mutant sequences harboring the Swedish (K670N, M671L), Dutch (E693Q, amino acid 22 of Aβ), and London (V717I, all in APP770 numbering) FAD mutations together with humanized Aβ sequence by homologous recombination (Figure 1A and B), so that expression of the mutant APP and human Aβ is subject to its endogenous physiological regulations. In the knock-in vector, a pair of loxP sites were inserted to flank the truncated exon16 (that stops before the mouse Aβ sequence) and the neo-cassette; followed by the mutant exon 16 encoding the Swedish mutation and humanized Aβ sequence, and the mutant exon 17 containing the Dutch and London mutations (Figure 1A). Mice containing the targeted allele were then crossed with a germline Cre-deleter [22] to remove the truncated exon 16 and the neo-cassette and to express APP with the Dutch/Swedish/London mutations and humanized Aβ sequence.

Preliminary analysis showed that, similar to the APP Swedish/London knock-in mice, the APP Dutch/Swedish/London mice develop minimal Aβ deposits in their life time, we accordingly bred both lines with the PS1 M146V knock-in mice [23-25] to facilitate the development of amyloid pathology within the aging time span of the mice. All subsequent studies were performed in the APP and PS1 M146V double homozygous background, listing the line with Swedish/London mutations and Dutch/Swedish/London as APP SL and APP DSL, respectively.

As expected, the APP DSL knock-in mice express similar levels of APP in comparison with that of wild-type mice and with an existing APP knock-in line expressing Swedish and London mutations and humanized Aβ sequence (APP SL) [20,21] (Figure 1C and quantified in 1D). Therefore, the APP DSL and APP SL knock-in lines are otherwise identical except the addition of the Dutch mutation in the APP DSL mice. .

### APP DSL knock-in mice exhibit accelerated amyloid pathology

**Figure 2:**
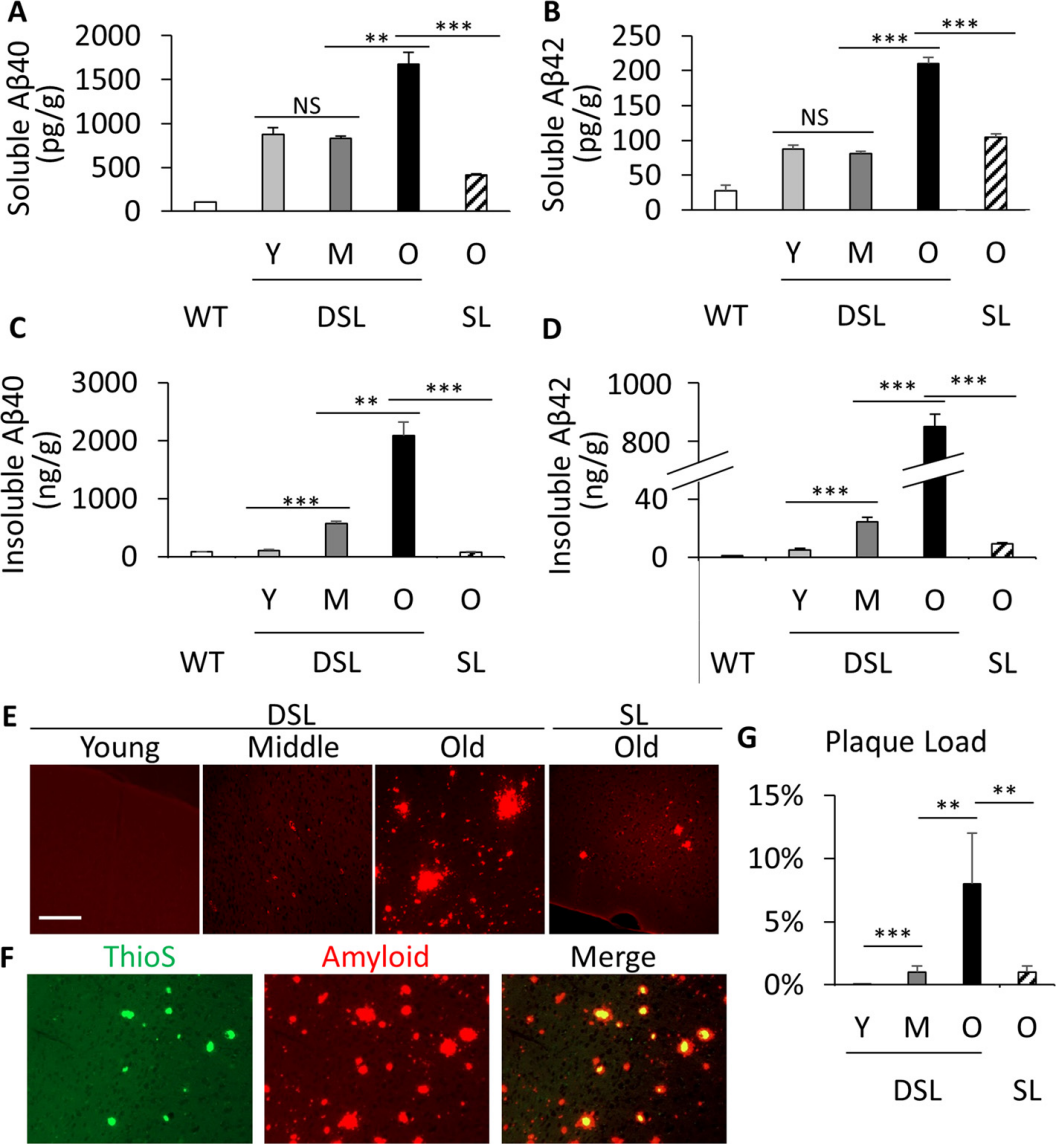
**Aβ levels and amyloid pathology in APP DSL and APP SL mice. A and B**. Sandwich ELISA analysis of Aβ1-40 (Aβ40, **A)** and Aβ1-42 (Aβ42, **B)** levels in Triton X100 soluble fractions of young (Y, ~ 6 month), middle (M, 10 ~ 12 month), and old (O, 18 ~ 20 month-double check Result section) of APP DSL and APP SL brain lystates. **C and D**. Aβ40 **(D)** and Aβ42 **(E)** levels in Triton X100 insoluble fractions of the corresponding age groups in **A & B**. WT: Wild type brain lysate, used as background control. Values of APP SL mice in only the old aged group was shown since the young and middle age groups were at background levels. **E**. upper panel, Aβ immunostaining using the anti- antibody showing age-dependent increases of parenchymal plaque in APP DSL knock-in mice. **F**. Fluorescent images demonstrate the consistence between the antibody staining and Thioflavin S (ThioS) staining. **G**. Quantification of percentage of plaques in whole cortical areas. N = 3 mice/genotype for young and old age group, and n = 5 mice for APP DSL middle age group. ***p < 0.001. Scale bar: 100 μm.

We performed sandwich ELISA to measure Aβ1-40 (Aβ40) and Aβ1-42 (Aβ42) concentrations in detergent soluble and insoluble fractions of brain lysates from young (Y, ~6 months), middle-age (M, 10 ~ 12 months), and old (O, 18 ~ 20 months) APP SL and APP DSL mice (Figure 2). Lysates from wild-type mice were used as a background control. We found that levels of Aβ40 and Aβ42 peptides in detergent-soluble fractions were comparable in young (light grey) and middle-age (dark grey) groups of APP DSL mice, but both were significantly elevated at old age (black bars Figure 2A and B). In contrast, a substantial elevation of insoluble Aβ40 and Aβ42 could already be detected in middle-aged APP DSL group compared to the young mice (Figure 2C and D), which is correlated with the beginning of amyloid deposition at this time frame (Figure 2E). Levels of insoluble Aβ, in particular Aβ42 (Figure 2D), increased sharply in old APP DSL mice, consistent with extensive amyloid deposition at this age (Figure 2E, 2F and quantified in 2G). These results indicate that the middle-age marks a critical stage when the APP DSL mice are no longer able to effectively maintain Aβ homeostasis, leading to elevated insoluble Aβ accumulation. The rise of soluble Aβ in addition to insoluble Aβ in old age likely reflects the further deterioration of the homeostasis. We did not isolate hippocampus for ELISA and plaque pathology due to the fact the plaque pathology was prominent only in the old age group of APP DSL mice in hippocampus, thus Aβ in total tissue lysate in ELISA and plaques in whole cortical area were used to indicate the progression of Aβ accumulation. In contrast to the APP DSL mice, Aβ levels and positive Aβ staining could only be detected in old APP SL mice (Figure 2, SL), but not in young and middle-age groups, when SL samples were prepared the same way as DSL samples. Therefore, introduction of the Dutch mutation leads to elevated steady-state Aβ levels across all ages and accelerated amyloid pathology.

### Anxiety-like behavior and cognitive impairment in APP DSL mice

**Figure 3:**
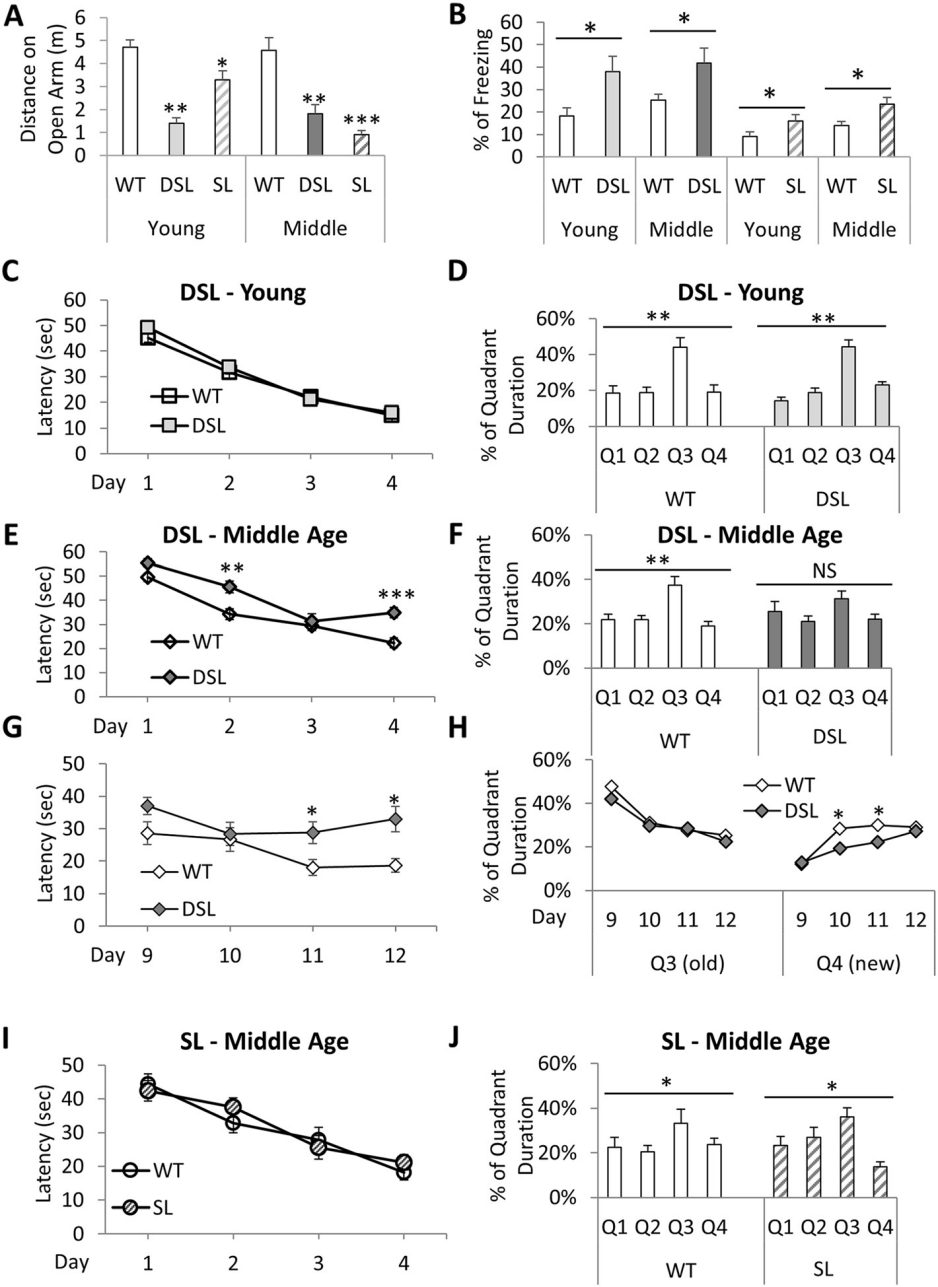
**Abnormal behaviors in knock-in mice.** Enhanced anxiety in both SL and DSL knock-in lines, but the memory impairments only in DSL mice starting at middle age. **A**. SL and DSL travels shorter distance on the open arms than wild type mice in elevated plus maze, indicating enhanced anxiety in these knock-in mice starting at young age. **B**. During the first 3 minutes when these mice were introduced to a new environment 24 hours after receiving a mild foot shock, both SL and DSL knock-in mice had higher percentage of time freezing in both young and middle aged group. **C-J**. Spatial learning and memory assays by Morris water maze (MWM). DSL exhibited normal learning and memory abilities when they are young (4-month old) **(C & D)**. But the middle-aged group were slow in reaching the hidden platform during learning **(E)** and showed memory deficits at elderly age (12-month old) **(F)**, in comparison with age and sex matched wild type control (WT). After additional day of training for DSL to learn the hidden platform tests, the platform was switched from Quadrant 3 (Q3) to Quadrant 4 (Q4) the following week to test reverse learning **(G and H)**. DSL mice lagged behind WT in learning the new position on Day 11 and 12 **(G)**, and in forming the new memory about the hidden platform on Day 10 and 11 **(H)**. In comparison with DSL, SL mice behaved normally in spatial learning and memory at the same aged period (12-month old) **(I & J)**. 10 ~ 18 male mice were used for each genotype at each age group. All mice used were novice to these behavior assays. NS, not significant; *P < 0.05, **P < 0.01, ***P<0.001.

We previously reported that the APP SL knock-in mice exhibit anxiety phenotypes starting at young age [20]. Analysis of young APP DSL mice revealed that these mice also display enhanced anxiety-like behaviors assayed by elevated plus maze with less distance traveled on the open arm than that of wild type mice (Figure 3A), and by conditioned fear assay with higher percentage of freezing time that was induced in a new environment (Figure 3B). These phenotypes persisted in middle-age animals. The enhanced freezing made it difficult to evaluate memory functions using the classical conditioned fear paradigm. We thus used the Morris water maze (MWM) to assess the spatial learning and memory abilities in APP DSL mice in young and middle-age groups and compared that with age-matched APP SL knock-in lines. APP DSL mice displayed normal spatial learning and memory at young age (Figure 3C and 3D). However, significant learning and memory deficits can be detected when APP DSL mice reached to middle age, as shown by longer latency to reach the hidden platform in the learning sessions (Figure 3E), and less duration in the targeted quadrant (Q3) during the memory probe test (Figure 3F). We subsequently investigated whether spatial related working memory is also affected in the APP DSL knock-in mice. Additional training was provided to allow the APP DSL reaching to the same level as the wild-type controls (Day 8) before they underwent reverse learning sessions (Day 9 ~ 12 in Figure 3G & H). During the following reverse-learning tests when the location of the hidden platform was switched from Quadrant 3 to Quadrant 4, the APP DSL mice extinguished the memory of the old location similarly as their wild type controls. However they formed the memory of the new location significantly slower than their wild-type controls (Figure 3H). The APP DSL mice were able to reach to a comparable level as wild type controls in the memory test at the last day of training, suggesting that their working memory deficits were at an early stage and could be corrected by intensive training (Day 12 in Figure 3H). Different from the APP DSL mice, the APP SL mice of the same age group demonstrated normal latency in locating the hidden platform in the training sessions as well as similar preference to the targeted quadrant in the probing test (Figure 3I and J). Old group of APP DSL mice exhibited the same anxiety behavior and learning and memory deficits as the middle age group, comparing with age and gender matched wild type controls (>18-month of age, n = 11 per genotype, Additional file 1). Since insoluble, but not soluble, Aβ were significantly increased in middle-aged APP DSL mice, the behavioral results indicate that the cognitive deficits are associated with insoluble Aβ accumulation. However, since ELISA was carried out using the whole hemisphere of the brain, we cannot exclude the possibility that there might be local changes of soluble Aβ levels in the middle aged group that were not observed from total tissue lysate.

### Age-dependent cerebral vascular phenotypes in APP DSL knock-in mice

**Figure 4:**
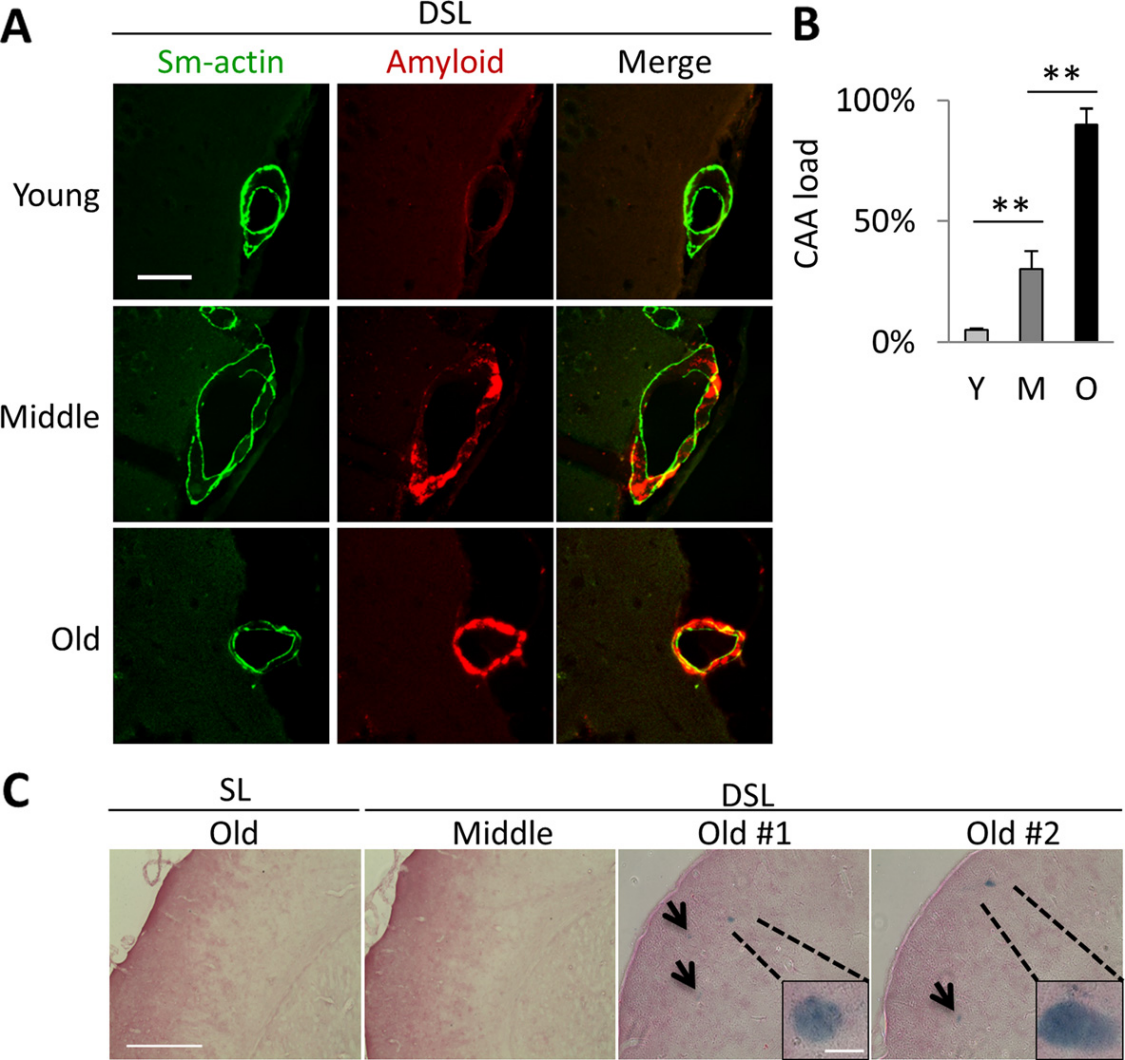
**Cerebral amyloid angiopathy and microhemorrhages in APP DSL mice. A**. Cerebral blood vessels (green) in DSL mice start to accumulate Aβ (red) around middle age (9 ~ 10 month of age), and became more extensively covered by amyloid at old age, as quantified in **B** (percent of smooth muscle-actin area covered by amyloid). Sm-actin, smooth muscle actin to mark blood vessels. **C**. Microhemorrhage in DSL at old age, as seen by Prussian blue staining. No blue sport detected on cortical area of any age of wild type or SL mice, nor in DSL young and middle age mice; but DSL old age mice (>17 month) frequently have microhemorrhage spots with diameters ranger from 10 ~ 50 μm. Average number of spots = 1.35 ± 0.46 per section, N = 10 sections/mouse from 5 animals. **P < 0.01. Scale bars: 50 μm in A, 400 μm in C, and 50 μm in C inset.

Since the Dutch mutation is known to cause CAA in humans, we next examined the CAA pathologies in APP DSL mice and compared these with the APP SL mice. Minimal CAA could be detected in APP DSL animals at young age (Figure 4A). However, prominent CAA could be observed when the mice reached to middle-age and became more severe as the mice aged (Figure 4A and quantified in 4B). No CAA could be detected in APP SL mice until they reached to terminal age (>28 month, Additional file 2). Therefore, inclusion of the Dutch mutation leads to a simultaneous increase of both vascular and parenchymal Aβ pathologies starting at middle-age.

**Figure 5:**
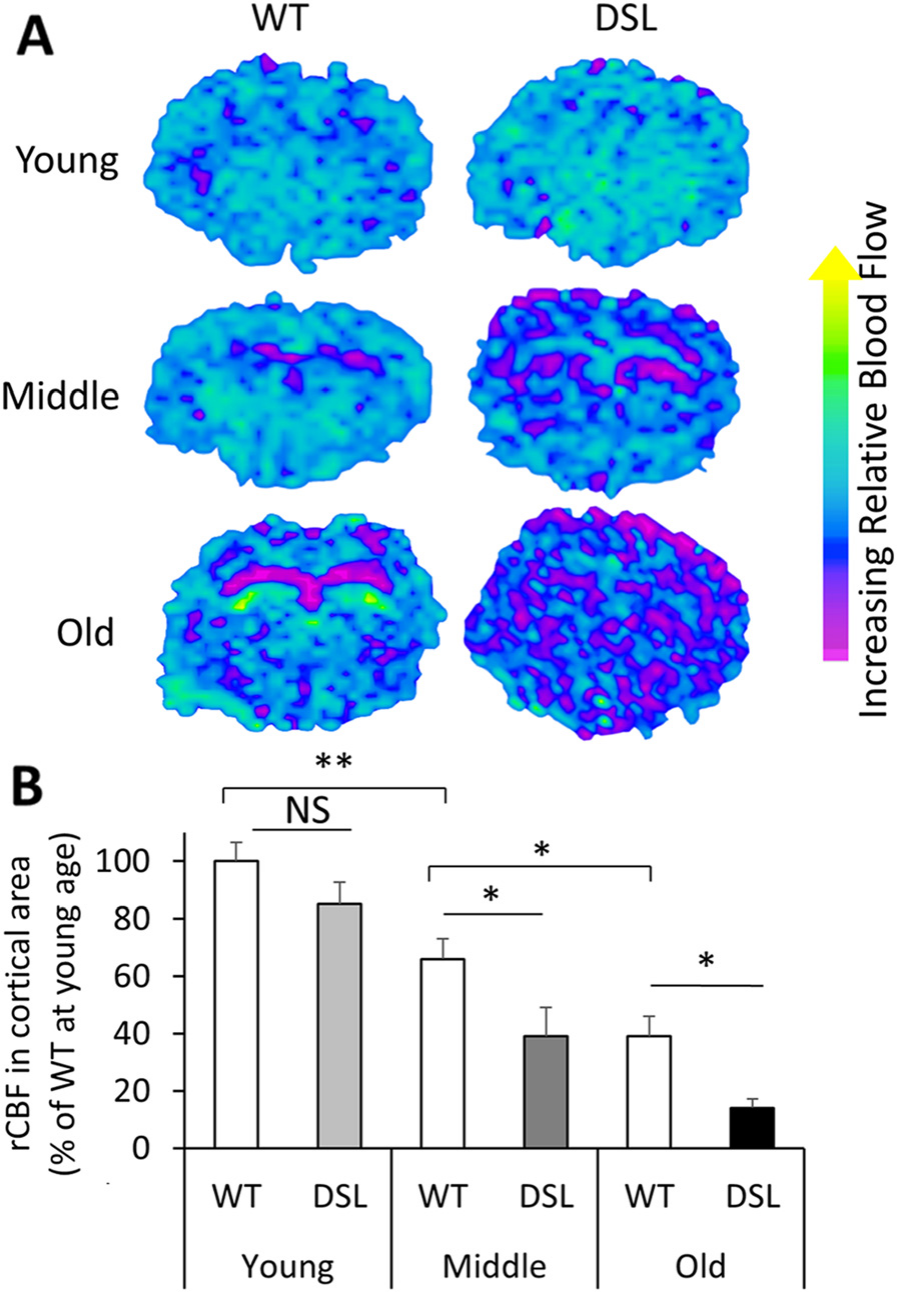
**Age-dependent reduction of rCBF in wild type and APP DSL mice measured by MRI. A**. Representative CBF maps for wild type (WT) and APP DSL knock-in mice at young, middle, and old ages. **B**. Quantification of CBF in the cortical area of MRI scans. N = 5-8 mice/genotype/age. NS, not significant; *P < 0.05, **P < 0.01.

Since Aβ accumulation surrounding blood vessels is known to increase the risk of intracranial hemorrhage and hemorrhagic stroke in human brains [26-28], we wanted to know whether similar pathology could also develop in APP DSL mice. We thus performed Prussian blue reaction to reveal iron released by hemosiderin in coronal sections of APP DSL mice and their age- and sex-matched wild-type controls and APP SL mice. No Prussian blue-positive signals could be spotted in cortical areas of wild-type or APP SL mice at any age (Figure 4C). While this was also the case in young and middle-age APP DSL mice, microhemorrhage became obvious in old APP DSL mice (Figure 4C, with average number of spots = 1.35 ± 0.46 per section, counted in 5 animals, 10 sections per animal). The microhemorrhages were almost exclusively located in the cortical region suggesting their association with amyloid buildup around cortical blood vessels. The fact that the onset of microhemorrhage is several months after the CAA supports the notion that CAA leads to the development of intracranial hemorrhage. Overall, the APP DSL knock-in mice recapitulate many of the pathological hallmarks associated with AD including age-dependent CAA and CAA-associated microhemorrhage.Because a prominent age-associated vascular Aβ pathology could be observed in the APP DSL mice, we asked whether CAA lead to changes in CBF in these mice. Using a MRI arterial spin labeling method, we measured relative cerebral blood flow (rCBF) in cortical regions of young, middle-age, and old wild-type and APP DSL mice (Figure 5). As expected, age-associated reduction of rCBF exists even in wild type mice (Figure 5B, open bars). Interestingly, the APP DSL mutant mice at young age already showed a mild reduction in rCBF compared to age-matched wild type controls, although this difference failed to reach statistical significance. As the mice aged, APP DSL mice develop much severe reduction in rCBF than their wild-type controls. We found that rCBF in APP DSL mutant was reduced to ~60% and ~40% of the wild-type controls at middle-age and old groups, respectively (Figure 5B), correlating with the severity of the CAA phenotypes (see Figure 4).

### Chronic manipulation of CBF results in global increase of CAA and plaque load

**Figure 6:**
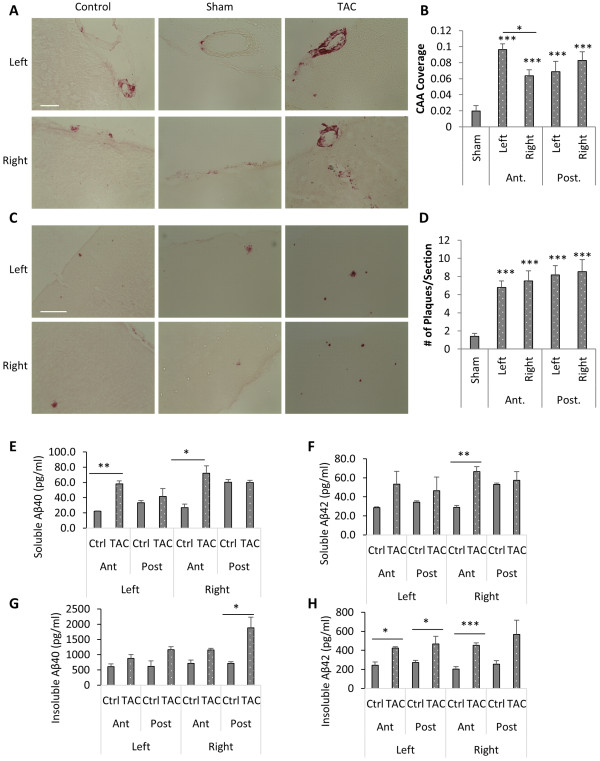
**Abnormal cerebral blood flow increases amyloid pathology. A. and C.** Representative images of CAA **(A)** and plaque **(C)** pathologies in anterior areas of control, sham-operated and transverse aortic constriction surgery (TAC)-operated APP DSL. TAC was performed on the left common carotid arteries of 6-month old APP DSL mice that terminated for analysis 3 months post-surgery. **B**. and **D**. Quantification of CAA **(B)** and plaque **(D)** load in left and right part of anterior (Ant.) or posterior (Post.) brain regions. Scale bars: 50 μm in A; 200 μm in C. N = 4 animals/group and 10 sections/animal. Coronal sections spanning from Bregma −2.58 ~ −0.58 mm were counted as anterior region;-0.94 ~ 3.64 mm were counted as posterior region. **E-H**, ELISA analysis of soluble **(E and F)** and insoluble **(G and H)** Aβ40 **(E and G)** and 42 **(F and H)** on anterior and posterior cortical areas in both left and right hemispheres. Each sample was measured in duplicates on ELISA plates. N = 3 animals/treatment. *P < 0.05, **P < 0.01, ***P < 0.001.

Having observed an exacerbating effect of CAA on age-dependent reduction of rCBF in the APP DSL knock-in mice, we set out to test if manipulation of blood flow could in turn affect Aβ pathology. We performed transverse aortic constriction surgery (TAC) on the left common carotid artery on 6 month-old APP DSL mice, a procedure that causes a substantial decrease of CBF on the anterior and medial regions of left side and a corresponding increase of CBF on the contralateral side of the same areas with minimal effects on the posterior regions. Non-invasive carotid Doppler ultrasound recording [29,30] confirmed the success of the surgery, with ratios of the peak flow velocity of the left carotid artery to the right side ranging from 1:6 ~ 1:12. We terminated the mice at 1.5 or 3 months after the TAC surgery when animals reached to 7.5 and 9 months old, respectively, to evaluate the state of CAA and Aβ deposits in the two hemispheres. We did not detect hemorrhages in these brains, indicating that there was likely no acute ischemic insult as might be expected with complete carotid artery occlusion. No appreciable Aβ staining can be detected in mice 1.5 months post-TAC, suggesting that the surgical procedure or attendant anesthesia did not significantly accelerate the onset of the cerebral or vascular Aβ pathology. However, when analyzed 3 months post-TAC surgery, the degree of both CAA (Figure 6A and quantified in B) and parenchymal Aβ pathologies (Figure 6C and quantified in D) were much higher in TAC-treated mice compared to sham-treated controls, the latter were indistinguishable to age-matched untreated mice. Interestingly and unexpectedly, increases in CAA and plaque deposits can be detected in the entire brain irrespective of left-right hemispheres or anterior-posterior regions. With the exception of CAA severity between the anterior left and right hemispheres, there were no statistically significant differences in all other comparisons (Figure 6B and D). Therefore, perturbation of CBF has a global effect on CAA and Aβ plaque pathology. This could be due to that either circulating amyloid-aggregation-prone factors were produced by regional changes of CBF or that regional manipulation of CBF results in global impairment of Aβ clearance and thus increased overall amyloid deposits.In order to quantify soluble and insoluble Aβ40 & 42 concentrations in different cortical areas in TAC animals 3 months post operation, we dissected cortical tissue into left/right anterior/posterior parts from Zn-fixed sucrose cryo-protected frozen TAC and control brains for ELISA quantification (Figure 6E-H). Since Zn-fixation and sucrose protect increased tissue weight, we didn’t convert the final readout into per gram of wet tissue as we did in Figure 2A-D. Instead, detergent soluble fractions were normalized to the samples’ protein concentrations (2.5 mg/ml), and insoluble fractions were prepared from re-solubilized pellets of guanidine solution from 10 mg/ml total tissue lysates (details in Materials and Methods section). We found that insoluble Aβ42 is increased in all regions in TAC animals, correlated with plaque and CAA load analysis, changes in the soluble Aβ40 and 42 are more prominent in the anterior region than the posterior region, corresponding to the blood flow changes in the anterior area of the brain. Again, no differences between left vs right were observed, suggesting aberrant blood flow in either directions are deleterious.

## Discussion

To investigate the involvement of neurovasculature in AD pathogenesis (for reviews: [6,31-33]), we created a new AD knock-in mouse model (APP DSL) that develops progressive CAA and amyloid pathology in the absence of transgene overexpression. Comparison of APP DSL and APP SL knock-in mice revealed that inclusion of the Dutch mutation results in not only prominent CAA but also accelerated plaque pathology; the onset of both coincides with the rise of insoluble Aβ levels in middle-aged animals. The Dutch mutation also triggers many age-related changes relevant to AD, including behavioral deficits, reduction of cerebral blood flow, and microhemorrhages. Surgical intervention of regional CBF changes in either directions resulted in a global increase on the effects of both CAA and plaque load.

Various transgenic mouse models expressing mutant *APP* with or without mutant *PSEN1* under the control of exogenous promoters have been created to model Aβ pathology. Analyses of these mice have provided invaluable insights into AD pathogenesis. However, to promote the development of amyloid pathology, these transgenic models typically overexpress mutant proteins several fold higher compared to the endogenous proteins. Considering the various physiological functions of APP and PS1 in the central nervous system, it has become increasingly clear that at least some of the biochemical and functional defects observed in these transgenic mice are due to mutant protein overexpression rather than pathological lesions (reviewed in [16] and see [17-19] for recent publications). The knock-in mouse models are particularly attractive as they express APP with FAD mutations and humanized Aβ under its endogenous temporal and spatial regulations throughout life, thus avoiding the confounding effects of ectopic transgene overexpression. Except the APP-NL-F and APP-NL-G-F mice reported recently [19], all other APP knock-in lines require crossing onto PS1 FAD background to facilitate Aβ development within a mouse’ life span. Inclusion of the Beyreuther/Iberian (I716F) mutation in APP-NL-F and APP-NL-G-F mice drives Aβ42 production at ~30× higher levels than Aβ40, thus avoiding the necessity of introducing PS1 mutation. However, the Aβ profiles expressed in these mice are distinctly different from that of typical AD. In comparison, although inclusion of the PS1 M146V knock-in allele is required for APP DSL mice to develop amyloid pathology, the Aβ40/42 ratio is comparable to what were observed in aged human brains and in mild cognitive impairment and AD cases [34-38]. Even though the APP DSL mice generate an Aβ variant that only exists in patients with HCHWA-D, these mice present prominent age-related CAA that is widespread in AD brains. This feature makes the model uniquely suitable for studying the role of cerebral vasculature in AD pathophysiology.

We are aware of the potential complications by including the PS1 M146V knock-in mutation and, accordingly, we used APP SL mice with the same PS1 M146V knock-in line as a control. Behavioral analysis showed that both APP DSL and APP SL knock-in mice develop anxiety-like phenotypes at a young age but only the APP DSL mice exhibit age-dependent spatial learning and memory impairment, supporting the notion that these cognitive and non-cognitive phenotypes are mediated by distinct pathways. This assessment is in agreement with our previous studies [20,39]. The onset of vascular and parenchymal Aβ deposits in the APP DSL knock-in mice coincides with cerebral hypoperfusion and spatial learning and memory deficits, thus recapitulates the prevalent early symptom in AD patients. It is still debatable regarding the cause or consequence between CAA and cerebral hypoperfusion in patients with both pathological symptoms. Our data suggest that higher steady-state levels of Aβ peptides, especially CAA, can be the causal factor for disturbance in CBF regulation, meanwhile, we found increased CAA in the setting of hyperperfusion as well.

It is known that anesthesia agents such as isoflurane at 1.6 ~ 1.8% concentration can cause vasodilation and increased cerebral blood flow [40]. Since CBF monitored by ASL MRI was scanned when mice were anesthetized by isoflurane within the concentration range, the severe reduction of CBF in the APP DSL mice detected, especially in old mice, could be a combination of chronic hypoperfusion in awakening state and a failure to response to isoflurane-induced CBF change in the knock-in animals. This is in consistent with published observations in AD patients and in AD transgenic mice models showing that the existence of impairments in autoregulation of cerebrovascular blood flow before the onset of the disease [41-44]. Therefore, the exacerbated age-dependent CBF reduction observed in APP DSL mice by MRI could indicate an impaired neurovascular coupling, resulting in a blunted response to vaso-regulative stimuli upon challenged states.

Our vessel constriction experiments demonstrate a direct modulation of CBF and Aβ deposition and a positive relationship between CAA and parenchymal Aβ. Contrary to a simple correlation between reduced CBF and increased Aβ burden, we observed global elevation of CAA and amyloid plaques in both hemispheres of mice undergoing aortic constriction surgery, although CAA appeared to be more sensitive to the regional changes of blood supply. TAC surgical procedure on the left common carotid artery is expected to result in blood flow changes in anterior and middle region of the brain with hypoperfusion on the left side and hyperperfusion on the right side, leaving blood flow at the posterior region intact according to the cerebral vasculature pattern [45]; this has been verified by fMRI after the surgery (Additional file 3). The differential changes in CBF on two hemispheres results in physiological changes in the same direction, which has been reported previously by Poulet et al. [46], in which elevated oxidative stress and BBB permeability were observed in young C57Bl/6 mice 8 weeks post-TAC. APP DSL mice might undergo similar changes, and in turn, increase overall amyloid pathology even though we didn’t detect BBB permeability in TAC-treated mice. Works from the same research group on C57Bl/6 J and receptor for advanced glycation end products (RAGE) knockout mice reported detecting Aβ deposits near cerebral vasculature in both cortex and hippocampus 8 weeks after the surgery, and reported that these amyloid depositions could be modulated by genetic or pharmacological inhibition of RAGE [47-49]. These results, on one hand are consistent with our observations that CBF changes in both directions can aggravated amyloid pathology, but on the other hand are different from our observations in that we did not observe amyloid deposit in wild type mice (C57Bl/6 J), nor can we detect amyloid deposits in our APP-DSL knock-in mice of the same young age group (3-month of age) 12 weeks post TAC surgery (data not shown). This discrepancy might result from differences in staining procedures as well as the difference in post-surgery time points. It is also intriguing that previous reports observed increasing amyloid depositions post-TAC in hippocampus [46-49], which we failed to see. Considering the fact that blood supply to hippocampus is from branches from vertebral arteries, CBF in the hippocampus would not be expected to be decreased by TAC. Since ischemia is known to trigger inflammation and production of reactive oxygen species (reviewed in [50]), it is possible that both regional hypoperfusion and hypertension are able to produce circulating factors (such as oxidative stress factors and inflammatory cytokines) that spread throughout the brain to regions where CBF has not been affected. These physiological changes from disturbances of cerebral hemodynamics in either direction, in turn, bring about an imbalance of Aβ production and clearance, and thus accelerates amyloid accumulation.

## Conclusions

In summary, the new APP DSL knock-in mouse model recapitulates critical features relevant to AD, including CAA, parenchymal Aβ plaques, cognitive impairments, cerebral hypoperfusion and microhemorrhage, in an age-dependent manner. These stages of pathological progression makes it an attractive model to decipher the relationship between vascular and parenchymal pathologies and to test the functional consequences by targeting these two pathologies in the absence of the confounding effects of transgene overexpression. Our findings support a model whereby regional reduction of CBF initiates a vicious cycle by causing widespread aggravation of amyloid pathology, which in turn leads to further deficits in CBF and exacerbation of Aβ and CAA pathology.

## Materials and methods

### Animals

All research and animal care procedures were approved by the Baylor College of Medicine Institutional Animal Care and Use Committee. PS1 M146V knock-in mice [23,24] and APP knock-in mice carrying Swedish and London mutations and humanized Aβ region (APP SL) were previously published. APP DSL knock-in mice with Dutch/Swedish/London mutations and humanized Aβ region was generated in the similar manner as previously published APP/hAβ/mutC [25], except that the Dutch mutation (E618Q, APP695 numbering) was incorporated by site-directed mutagenesis when constructing the gene-targeting vector. Briefly, exons 16 and 17 of mouse *APP* were replaced by the mutant construct from the knock-in vector by homologous recombination, in which a pair of loxP sites flanked truncated exon 16 and neo-cassette, followed by mutant exon 16 with Swedish mutation (KM595-596NL) and humanized Aβ sequence (G601R, F606Y, and R609H), and then mutant exon 17 carrying Dutch mutation (E618Q) and London mutation (V642I) (Figure 1A). Genotyping was done by PCR using primers:

HL033, 5′-GTAATGCCTGTGTGGCCAAACACATG-3′ and

HL037, 5′-AAGTAATGGATTTGTTCTCCCAGGTCG-3′, which amplifies the loxP site. Expected PCR product from the wild type allele is 230 bp, and from the targeted allele is 270 bp.

The mice were then crossed with a germline Cre-deleter [22] to remove the truncated exon 16 and the neo-cassette and to produce APP with Dutch/Swedish/London mutations and humanized Aβ sequence. Crossing with PS1 M146V knock-in mice generate APP/PS1 homozygous double knock-in mice.

### Sandwich ELISA

For Figure 2A-D, brain halves were weighted and homogenized in 4× (w/v) homogenization buffer composed of PBS, 1% Triton X100 and complete protease inhibitor cocktail (Roche). Total brain lysates were centrifuged at 100,000x g at 4°C for 1 hour and supernatants were diluted in half to be applied to Aβ1-40 and Aβ1-42 ELISA kit (Invitrogen, #KHB3481 and #KHB3544) following the company’s protocol. The resulting pellets (insoluble fractions) were resuspended in 5 M guanidine HCl/50 mM Tris HCl, pH8.0, rotated at room temperature for 4 hours, and centrifuged at 20,000×g for 20 minutes. Supernatants from the last step centrifugation were diluted 500 ~ 2000 fold before applying to the ELISA kits. Each sample was analyzed in duplicate. Three to five male animals were used for each genotype at each age group.For Figure 6E-H, 9-month-old male mice (with or without transverse aortic constriction surgery (TAC) at 6 months) were perfused and fixed using Zn-fixatives. The retrieved brains were cryoprotected by soaking in 30% sucrose for 36 hours and frozen in −80°C before ELISA experiment. To obtain cortical tissue for left/right anterior/posterior region, mouse brain was put into acrylic 1 mm adult mouse brain matrix for coronal slices to remove olfactory bulbs and cerebella. Cutting in the mid-line separates left vs right half of the brain, cutting in the 5 mm slot separates anterior and posterior region. Corresponding parts of cortex were then peeled off and detergent soluble and insoluble lysate were prepared as mentioned in the above paragraph. Due to Zn-fixation and sucrose cryoprotection, the tissue weighted heavier than un-perfused tissue used for Figure 2A-D, rendered it impossible to deduce comparable Aβ concentration in per gram of wet tissue as before. Thus, ELISA results for soluble samples were normalized to the protein concentration of 2.5 mg/ml. For insoluble fractions, 300 ul of 10 mg/ml of total tissue lysate were centrifuged for 1 hour at 100,000×g 4°C; resulting pellets were re-solubilized in 200 ul of guanidine solution as in the above paragraph, and diluted 20× before applying to ELISA plates. All data in this group were presented as pg/ml instead of converting to pg/g of wet tissue. All samples were analyzed in duplicates on ELISA plates, and 3 male animals were used for each group of treatment.

### Immunofluorescence, immunohistochemistry staining (PFA or Zinc fixed), and amyloid load quantification

Antibody staining on paraffin-embedded brain sections was performed as described [24]. In particular, paraformaldehyde (PFA) perfused brains were cut into 10 μm paraffin sections. The sections were deparaffinized in xylene, then rinsed with series of ethanol and water mixture. Antigen-retrieval was done by incubating sections with 70% formic acid for 6 minutes. Endogenous peroxidase activity was quenched by incubating the slides in 0.3% H_2_O_2_ for 30 minutes. The slides were rinsed with Tris-buffered saline (TBS; 50 mM Tris-Cl, pH 7.5 and 250 mM NaCl). Nonspecific epitopes were blocked for 30 minutes with 3% normal goat serum, 0.4% Triton X-100 in TBS. Primary antibodies against smooth muscle actin (Sigma, A2547) and/or Aβ (Cell Signaling, #2454) were diluted 1:1000 in blocking buffer, and incubated overnight at 4 ° C in a humid chamber. Sections were then washed 3 x 5 minutes each in TBS and incubated with anti-mouse-Alexa 488 and/or anti-rabbit-Alexa 555 secondary antibodies at room temperature for 1 ~ 2 hours. Pictures were taken with a Leica TCS SPE microscope and the images were processed with ImageJ (National Institute of Health). Mice underwent aortic banding surgery were fixed in a similar manner but using Zinc-fixation [51] (0.1 M Tris–HCl pH7.4, 0.05% Calcium acetate, 0.5% Zinc acetate, 0.5% Zinc chloride) instead of PFA. Immunohistochemistry on Zn-fixed brain sections were done using alkaline-phosphatase standard method (Vector Laboratories, AK-5000) and corresponding alkaline phosphatase substrate kit (Vector Laboratories, SK-5100).

### Prussian blue staining for microhemorrhage

Cryosections of WT, SL, and APP DSL mice were mounted on glass slides and dried overnight. Slides were immersed in freshly mixed 4% Potassium Ferrocyanide with 4% of hydrochloric acid, and microwaved for 30 seconds. Then rinsed in tap water for 3×10 minutes. Next, slides were counter stained using nuclear fast red at room temperature for 5 minutes, rinsed under running tap water for 1 minutes, dehydrated quickly in 95% and 100% ethanol, cleared in xylene, covered by mounting medium and coverslips for imaging. 3 ~ 4 male animals were used for each genotype at old age group as well as APP DSL in middle age group.

### Behavioral assays

Mice were housed in a 12 hour light/12 hour dark cycle. All behavior tests were done on naïve male mice at either young (4 ~ 6 month old) or middle (12 ~ 13 month old) ages. 10 ~ 18 male animals were used for each genotype at each age group. Mice were tested in elevated plus maze first, conditioned fear experiment two days after, and the Morris water maze one week later.

### Elevated Plus Maze (EPM)

The testing room was at 700 LUX illumination, with 60 dB white noise. Mice were brought to the room in their home cages and allowed to acclimate for 1 hour before starting the test. The testing apparatus is a plus-shaped platform made of white plastics, elevated 50 cm above the ground, with each arm 30×5 cm. Extended from a 5×5 cm center area are two opposite high-walled enclosed arms and two open arms (with a shallow rim along the side). The mouse being tested was placed in the center area and allowed to explore the plus maze for 10 minutes. Entrances, duration, and distance traveled in each region were recorded by a camera connected to an automated ANY-maze tracking system (Version 4.50; Stoelting, Co., Wood Dale, IL, USA). The platform was cleaned with 70% isopropanol after each trial. All mice were novel to the test, and EPM is the first behavior test performed on these mice.

### Conditioned Fear (CF)

Mice were transported to the holding room in their home cages 30 minutes prior to the test on the training and test days. On the first day (training sessions), each mouse was transferred to the testing room, placed in the isopropanol-cleaned chamber for 2 minutes, and then received a mild foot shock (2 seconds, 0.7 mA) delivered from the grid floor each time after hearing a 30-second tone (conditioned stimulus); 2 minutes later, the tone and the shock would repeat again, and the mouse would be removed from the chamber and transferred back to the holding room. 24 hours after the training session, mice were first tested for freezing behaviors in the same chamber without the tone stimulus (identical context). 2 hours later, mice were tested for freezing behavior in an altered environment. Each mouse was transported by the same experimenter to the same experiment room, but into the 70% ethanol-cleaned chamber with vanilla smell (different odor cue), triangle space (different testing space), and smooth white plastic floor (different tactile cue), for a total of 6 minutes. The first 3 minutes were quiet and the last 3 minutes the tone was played. Movements of the mouse were recorded by a video camera and analyzed by FreezeFrame software (Version 2; San Diego Instruments).

### Morris water maze assay

Morris water maze tests were performed as previously published [52-54]. Briefly, mice were trained to locate a square platform (9 × 9 cm) hidden in quadrant 3 of a circular water pool within 60 seconds. In the learning sessions, each mouse was trained in total of 32 trials for four consecutive days. Each day, each mouse received 2 blocks of training, and in each block, the mouse was trained for 4 successive trials. At the end of the 32 trials, the mouse was subjected to a probe test during which the platform was removed and each mouse would swim for 60 seconds to search for the platform. APP DSL/PS1 double knock-in mice, together with their wild-type control group, received a simplified training of 8 trials on Day 5, in which mice were returned to its holding cage after each swimming trial. The mice underwent reverse learning sessions the following week, in which the location of the hidden platform was moved from quadrant 3 to quadrant 4. Mice were trained in 4 trials each day for another four consecutive days. On Day 9, before the reverse learning session, mice were exposed to a probe test of 60 seconds, in which the platform was removed for the 60-second probing period but placed back at its old location at the end of the session (to minimize the extinction effect of probe run). Probe tests on Day 10 ~ 12 were done after the training sessions, and the hidden platform was put back into the pool at the new location at the end of each test. For all the swimming sessions, the training/testing mouse was rested on the platform for 10 seconds before it was submitted to the next trial or transferred back to its holding cage. The time for mice to locate the hidden platform during learning and reverse learning sessions (escape latency), as well as the time they spent in each quadrant during the probe tests (quadrant duration) were recorded by Ethovision tracking system (Noldus Information Technologies).

### Magnetic Resonance Imaging (MRI)

All mice were initially anesthetized with 5% isoflurane in oxygen and then transferred to an animal holder within the magnet where they were maintained on 1.5-2% isoflurane in oxygen during imaging. Respiration rate was monitored using a respiration pad (Small Animal Instruments) and maintained at approximately 30–35 breaths per minute to minimize motion artifacts during imaging. Body temperature was monitored with a rectal temperature probe and was maintained at a constant 37°C using an air heating system (Small Animal Instruments) throughout the relative cerebral blood flow (rCBF) measurement. MRI imaging was carried out utilizing a 9.4 T, Bruker Avance Biospec Spectrometer with a 21 cm horizontal bore and a 35 mm volume resonator (Bruker BioSpin).

A slice of interest within the brain was selected using setup scans aligned to where the rostral segment of the hippocampus first appears. The rCBF was measured using an arterial spin labeling (ASL) MRI imaging protocol. A flow alternating inversion recovery-echo planar imaging protocol with the following parameters was utilized: TR = 7555.38; TE = 16.73; NA = 1; matrix = 64×64; field of view = 1.5cmx1.5 cm; slice thickness = 1.5 mm. Both a selective and nonselective ASL image was acquired and the absolute blood flow was then calculated within two regions of interest within the cortex using the formula rCBF = λ(1/T1selective – 1/T1global) where λ is the blood brain partition coefficient which in the mouse brain is equivalent to 0.9. Five male mice per genotype of young and old groups, and 8 per genotype of middle aged group were tested in this experiment.

### Transverse Aortic Constriction surgery (TAC)

Transverse aortic constriction and Doppler evaluation of the blood flow were performed in mice using the method described in previous publications [55]. In brief, to produce a transverse aortic constriction, mice were anesthetized with an initial dose of 5% Isoflurane and maintained on 2% isoflurane via inhalation, and were placed on a heating pad at 38.6°C to maintain body temperature during the surgical procedure. The animal was placed supine and endotracheal intubation was performed. The endotracheal tube was connected to a Harvard volume-cycled rodent ventilator cycling at ≈ 150 breaths/minutes and with sufficient volume for adequate expansion of the lungs, with a tidal volume of approximately 0.20 -0.25 ml. Oxygen (100%) and maintenance dose of 1–1.5% isoflurane was supplied to the inflow of the ventilator during operation. Under a microscope, the chest cavity was opened and the aortic arch was exposed after deflection of the thymus. After the transverse aorta was isolated, a 7–0 nylon suture was placed around the aorta between the origins of the innominate and left carotid arteries. A 27-gauge (0.4 mm diameter) needle was placed against and tied to the aorta with the suture. Then the needle was removed promptly to yield a constriction with a diameter approximately equal to that of the needle, and produce a 60-80% constriction. In sham-operated mice, the suture was placed around the aorta but not tightened. The chest wall was closed and the mice were allowed to recover from anesthesia under oxygen and a heat lamp to maintain body temperature. Doppler velocity was measured in the right and left carotid arteries to confirm that the blood flow velocity was reduced on the left and increased on the right side and the severity of the constriction is assessed by the ratio of the left to right peak carotid flow velocities [55]. Brains from 4 male mice per treatment group were used for paraffin section/immunohistochemistry analysis, and 3 male mice per genotype for 3 month old group were used in Aβ40 and 42 ELISA analysis.

### Statistics analysis

All raw data (from ImageJ, ELISA assay, and behavior tests) were tabulated in Microsoft Excel. Statistical analyses were performed using student *t*-test in Excel, or two-way ANOVA in GraphPad Prism4 (GraphPad Software, http://www.graphpad.com). Data were presented as average ± SEM (standard error of mean). NS stands for no significance; *, p < 0.05; **, p < 0.01; and ***, p < 0.001. For Morris Water Maze assays, escape latency and distance traveled data were analyzed using a two-way (genotype × trial block) ANOVA with repeated measures. Search data from the probe trial were analyzed by individual one-way ANOVA and Newman-Keuls post comparison tests. One-way ANOVA was used to compare the search time and platform crossing data for the training quadrant only between wild-type and mutant mice.

## Abbreviations

AD: Alzheimer’s disease; APP: Amyloid β (A4) precursor protein; ASL: Arterial spin labeling; BBB: Blood–brain barrier; CAA: Cerebral amyloid angiopathy; CBF: Cerebral blood flow; DSL: Dutch, Swedish, and London mutation; ELISA: Enzyme-linked immunosorbent assay; EPM: Elevated plus maze; FAD: Familiar Alzheimer’s disease; HCHWA-D: Hereditary cerebral hemorrhage with amyloidosis of Dutch type; MRI: Magnetic resonance imaging; MWM: Morris water maze; PS1 or PSEN1: Presenilin-1; PSEN2: Presenilin-2; RAGE: Receptor for advanced glycation end products; SL: Swedish and London mutations; sm-actin: Smooth muscle actin; TAC: Transverse aortic constriction; WT: Wild type.

## Competing interests

The authors declare no competing financial interests.

## Authors’ contributions

APP-DSL mouse line is generated by HL with help from KT and REH; mouse behavior experiments were performed by HL and QG; western blot analysis, ELISA of Aβ, immunofluorescent, and immunohistochemistry are done by HL with help from QG; fMRI scan and analysis are done by TI and RGP; transverse aortic constriction surgery and Doppler flow velocity tests are performed in GET’s lab under the guidance of GET; amyloid analysis after surgery is performed by HL; microhemorrhage analysis done by HL with help from VAP. Manuscript was written by HL and HZ. All authors read and approved the final manuscript.

## Supplementary Material

Additional file 1 APP DSL mice at old age persist with anxiety phenotype and deficits in learning and memory. Click here for file

Additional file 2 APP SL mice over 28 month old have severe parenchymal plaques and mild CAA. Click here for file

Additional file 3 MRI scan images of anterior and posterior region of a brain 1 week post-TAC surgery, showing hypoperfusion on the left hemisphere only at the anterior region of the brain. Click here for file
