# Supplementary material for: Vascular and parenchymal amyloid pathology in an Alzheimer disease knock-in mouse model: interplay with cerebral blood flow
Source: Mol Neurodegener. 2014 Aug 9;9:28. doi: 10.1186/1750-1326-9-28 (PMC4132280; doi:10.1186/1750-1326-9-28)
Supplement: Additional file 1 — APP DSL mice at old age persist with anxiety phenotype and deficits in learning and memory. [file 1750-1326-9-28-S1.pdf]

Additional file 1: APP DSL mice at old age persist with anxiety phenotype and deficits in learning and memory.

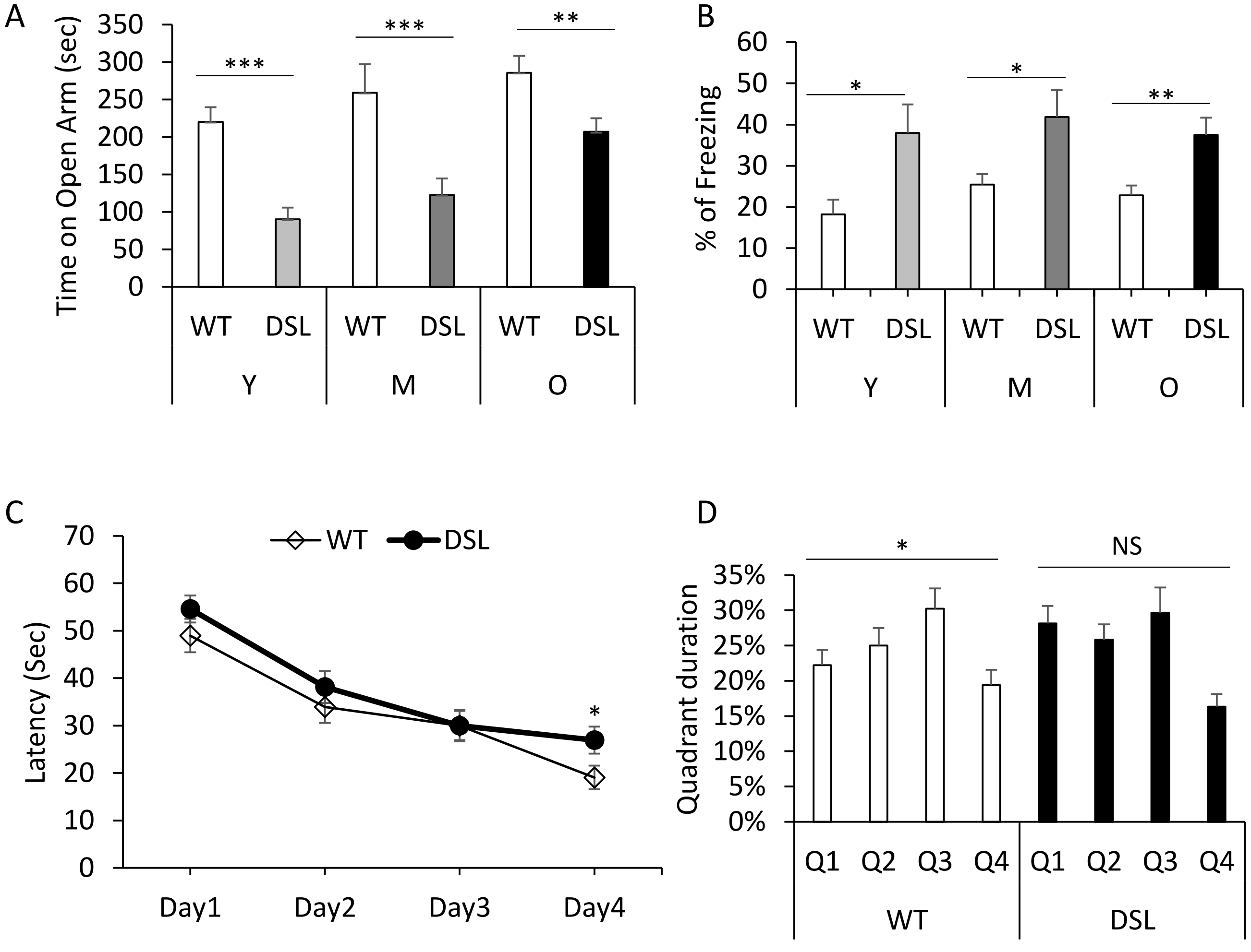

Additional file 1: Mouse behavior assays showing anxiety phenotype and memory deficits persisted till old age. A. All age groups of DSL mice spent less time on the open arm on elevated plus maze. B. All age group of DSL mice freeze more when entering a new environment after food shock. C. 18 month group DSL mice took longer time than wild type to reach the hidden platform on Day 4 of Morris water maze training. D. 18-month old DSL mice do not distinguish the targeted quadrant (Q3) as well as the wild type in memory probe test. N>11 per genotype per age group. Data presented are average±s.e.m.
