# Supplementary material for: Vascular and parenchymal amyloid pathology in an Alzheimer disease knock-in mouse model: interplay with cerebral blood flow
Source: Mol Neurodegener. 2014 Aug 9;9:28. doi: 10.1186/1750-1326-9-28 (PMC4132280; doi:10.1186/1750-1326-9-28)
Supplement: Additional file 2 — APP SL mice over 28 month old have severe parenchymal plaques and mild CAA. [file 1750-1326-9-28-S2.pdf]

Additional file 2: APP SL mice over 28 month old have severe parenchymal plaques and mild CAA.

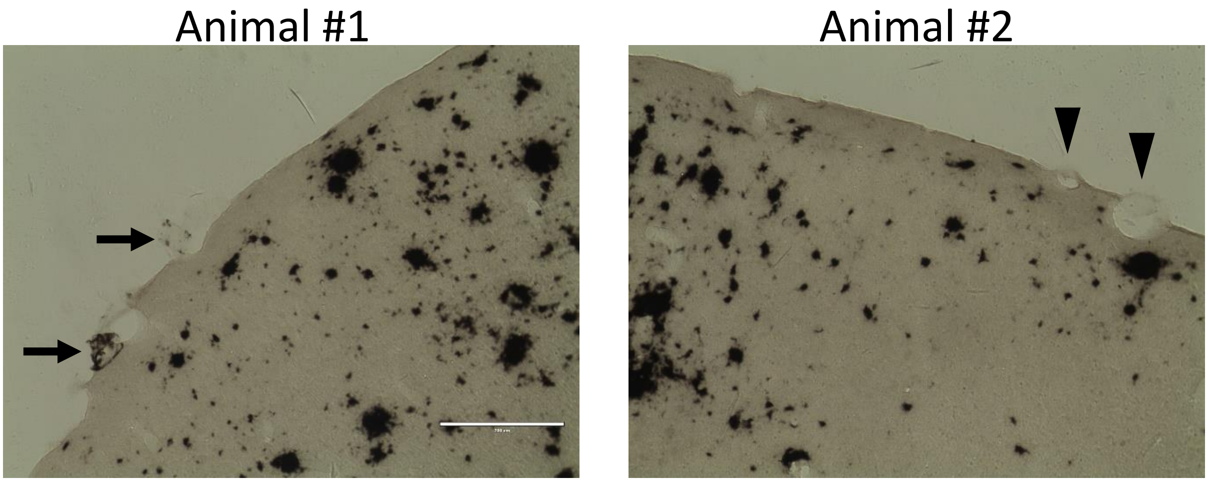

Additional file 2. Immunohistochemistry using antibody against amyloid showing APP SL mice at 30-month of age when severe plaque pathology appears, but still with very mild CAA on blood vessels. Arrows in the left image indicates blood vessels with CAA, and arrow heads in the right image point to blood vessels still have no sign of amyloid. Scale bar: 200µm.
