# Supplementary material for: Vascular and parenchymal amyloid pathology in an Alzheimer disease knock-in mouse model: interplay with cerebral blood flow
Source: Mol Neurodegener. 2014 Aug 9;9:28. doi: 10.1186/1750-1326-9-28 (PMC4132280; doi:10.1186/1750-1326-9-28)
Supplement: Additional file 3 — MRI scan images of anterior and posterior region of a brain 1 week post-TAC surgery, showing hypoperfusion on the left hemisphere only at the anterior region of the brain. [file 1750-1326-9-28-S3.pdf]

Additional file 3: MRI scan images of anterior and posterior region of a brain 1 week post-TAC surgery, showing hypoperfusion on the left hemisphere only at the anterior region of the brain.

Anterior

Posterior

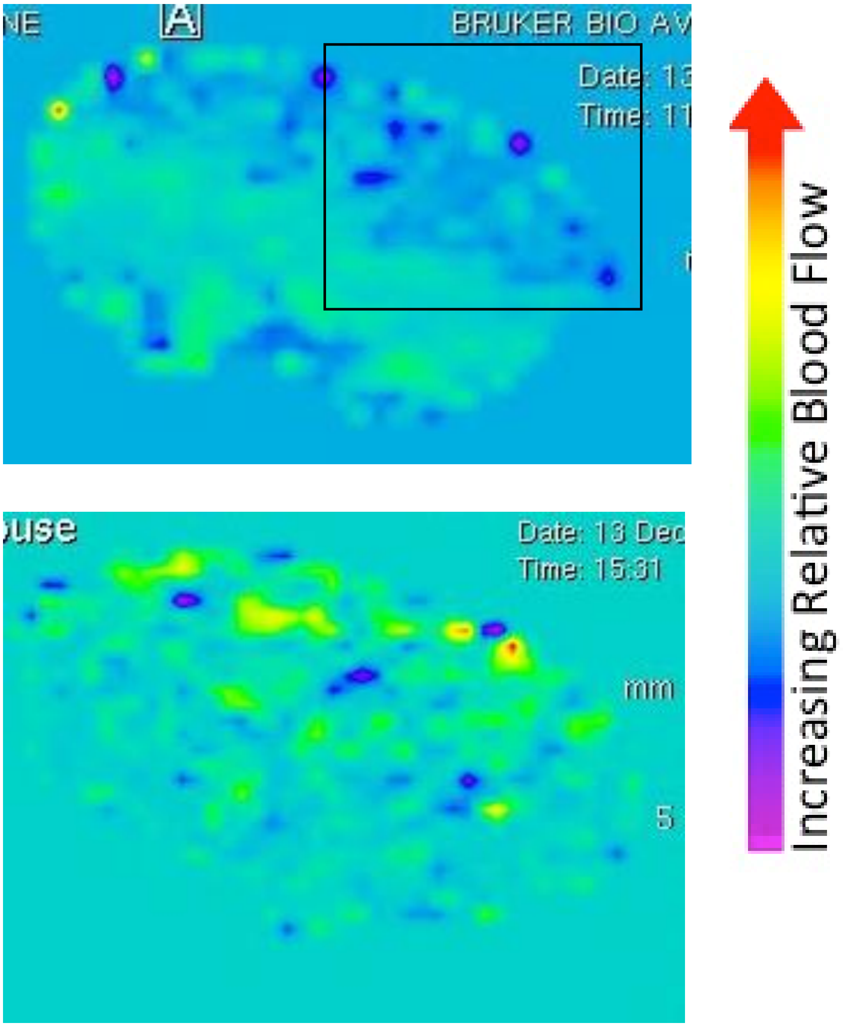

Additional file 3: MRI scan images of anterior and posterior region of a brain 1 week post-TAC surgery. Doppler velocity of left to right ratio on carotid arteries is 1:9 on this mouse. The black rectangle in the upper images frames the hypoperfused area in the anterior region of the brain.
